# Perceptual sensory attenuation in chronic pain subjects and healthy controls

**DOI:** 10.1038/s41598-022-13175-4

**Published:** 2022-05-27

**Authors:** David McNaughton, Alissa Beath, Julia Hush, Michael Jones

**Affiliations:** 1 School of Psychological Sciences, Macquarie University, Balaclava Road, Sydney, Australia; 2 Department of Health Services, Macquarie University, Sydney, Australia

**Keywords:** Neuroscience, Physiology, Psychology, Medical research, Pathogenesis, Signs and symptoms, Engineering

## Abstract

We investigated whether sensory attenuation (or failure of) might be an explanation for heightened pain perceptions in individuals with chronic pain. N = 131 (50% chronic pain) individuals underwent a single experimental session, which included the force-matching task and several self-reported symptom and psychological measures. Individuals matched a force delivered to their finger, either by pressing directly on their own finger with their other hand (direct) or by using potentiometer to control the force through a torque motor (slider). All participants overestimated the target force in the direct condition reflecting the sensory attenuation phenomenon. No differences in the magnitude of sensory attenuation between chronic pain and control groups were observed (direct: Z = − 0.90, p = 0.37 and slider: Z = − 1.41, p = 0.16). An increased variance of sensory attenuation was observed in chronic pain individuals (direct: F(1, 129) = 7.22, p = 0.008 and slider: F(1, 129), p = 0.05). Performance in the slider condition was correlated with depressive symptoms (*r* = − 0.24, p = 0.05), high symptom count (*r* = − 0.25, p = 0.04) and positive affect (*r* = 0.28, p = 0.02). These were only identified in the chronic pain individuals. Overall, our findings reveal no clear differences in the magnitude of sensory attenuation between groups. Future research is needed to determine the relevance of sensory attenuation in neuro-cognitive models related to pain perception.

## Introduction

Chronic pain is commonly conceptualised within a biopsychosocial framework; in which a complex interaction of biological, psychological, and social factors generate a multidimensional symptom experience^1^. One critique of this approach is the lack of detailed causal pathways to explain mind–body interactions^2^, which has led to advances in emerging models of symptom perception such as those based on Bayesian approaches, described as Predictive Processing models^3^. While contemporary biomedical theories describe perception as a bottom-up interpretation of sensory information, emerging Bayesian models suggest that perception can be understood through neurological processes of prediction, based on the integration of sensory inputs, prior experiences, and contextual cues^4,5^. This model of Predictive Processing is a useful conceptualisation for the relationship between expectations, attentional biases, and physical symptoms, and may also further enlighten poorly understood conditions, such as those with chronic pain. However, there is currently a lack of consistent experimental evidence to support this model in such conditions.

A key component of Predictive Processing is that perception is not solely driven by sensory evidence, but also by the brain’s inferences, which are continuously refined by sensory evidence^6^. Within this model, descending predictions from higher levels of neuronal hierarchies (termed priors) are compared with lower-level representations to form prediction errors^7^. These prediction errors, or mismatch in signal, are passed back up the hierarchy to update higher cortical representations. A fundamental aspect of this system is to minimise prediction error, and this may be achieved by (1) updating prior perceptions of the cause of sensory information or (2) altering the sensory evidence so that it conforms to one’s predictions via weighting (or selectively filtering) the sensory evidence^8^. An example of latter is the integration of sensory evidence with predictive signals from forward motor models which lead to sensory attenuation^9^.

During human movement, internal models in the central nervous system predict the outcome of actions via an efference copy of the motor command. If the predicted sensation associated with that movement (the “efference copy” signal) corresponds with the incoming sensory signals produced by the motor output, a sense of agency is experienced^10^. In contrast, a mismatch between prediction and sensation suggests an external event has occurred and the individual does not experience agency. A key neural component of this process is that the predicted sensory stimuli associated with movement are compared with the actual sensory feedback, which partially cancels out sensory consequences of self-generated movement^11,12^. This process is termed perceptual sensory attenuation and ensures the system is robust in the face of delays or noise associated with sensory processing^13^. Sensory attenuation helps explain the ineffectiveness of behaviours such as tickling ourselves^14^ and attenuation of sensory effects of ones actions has also been reported in auditory and visual perception^15^.

A related, yet distinct, parallel can be drawn from attentional bias literature, in which the altered cognitive processing of has been identified in some individuals with chronic pain, and this may influence the primary appraisal of environmental stimuli^16^. This attentional bias related to emotional processing is proposed to be related to the pathophysiology of chronic pain, however the exact mechanisms that explain unconscious attribution of attention remain unclear^17^. In the context of this study, we investigated whether the unconscious integration of sensory signals with forward motor models, leading to sensory attenuation are altered in chronic pain. It has been argued that sensory attenuation is essentially the process of turning up (or down) the gain of a specific sensory channel, through selectively weighting sensory evidence^18^. This may be an underlying mechanism which helps explain an increase in body focussed attention described in the attentional bias literature. Specifically reflecting a reduction in the ability to attenuate the sensory consequences of one’s own actions^19^.

An experimental paradigm to quantitively measure the phenomenon of sensory attenuation is the force-matching task^20^. In this task, subjects are asked to match a force delivered to their finger, either by pressing directly on their own finger with their other hand (known as the direct condition) or by controlling the device using an external potentiometer to control the force indirectly through a torque motor (known as the slider condition). Previous research has shown that healthy people consistently generate a greater force and tend to overestimate the force in the direct condition when compared to the slider condition^9,21,22^. The excess force produced in the direct condition reflects the sensory attenuation phenomenon. A failure of sensory attenuation may result in false inferences about the cause of self-generated acts, and has been suggested to help explain symptoms of schizophrenia^23^ and functional motor syndromes^19^. In saying this, we do not believe the aetiologies between chronic pain, schizophrenia or functional motor symptoms are similar. They have distinct primary causes, through a potentially common or overlapping neurobiological pathway.

Previous research has suggested pain may be perceived in the presence of non-noxious sensory input, in part, due to a failure of sensory attenuation^24^. Sensory attenuation potentially provides a mechanism in which in the presence of rigid pain-related priors, subsequent and selective weighting (or filtering) of sensory evidence may occur^3^. This has been concluded with limited experimental evidence. It is the purpose of the current study to investigate the sensory attenuation phenomena in chronic pain measured through the force-matching task. Specifically operationalising sensory attenuation as the reduction in the perception of the afferent input of a self-produced tactile sensation due to the central cancellation of the reafferent signal by the efference copy of the motor command to produce the action^25^. In light of this, reduced sensory attenuation may be viewed as a central neurological process which may be shared amongst individuals with different aetiologies or locations of chronic pain, similar to other related, yet distinct processes such as central sensitisation (e.g. allodynia and hyperalgesia) or deficits in proprioception and interoception^26–28^.

It is the aim of the current study to investigate the sensory attenuation phenomenon in individuals with chronic pain and identify whether there is evidence of correlation with symptom and/or psychological measures. We specifically hypothesise chronic pain individuals experience reduced sensory attenuation, reflecting an altered weighting of sensory information as a potential mechanism for underlying attentional biases. To test this, we conducted the force-matching task in subjects with and without chronic pain, using a bespoke force-matching device^29^. To our knowledge this paradigm has not been explicitly tested in a chronic pain sample, and in the process of testing our a-priori hypotheses we made observations which led to additional research questions concerning inter-individual variance in sensory attenuation.

## Methods

### Subjects

Two groups were recruited for the study: a chronic pain group and matched healthy control group. All participants were right-handed, above the age of 18 and without any neurological, respiratory, or psychiatric illness (such as schizophrenia). Eligibility criteria for the chronic pain group were: the presence of persistent somatic pain for 3 months or more, for which treatment had been sought. The eligibility criterion for the control group was that they currently experienced no pain. Informed consent was obtained from all participants, and this research was performed in accordance with the declaration of Helsinki.

Chronic pain and control subjects were recruited via advertisements distributed throughout Macquarie University, Sydney, Australia or through screening undergraduate psychology students at Macquarie University. All subjects took part in a single experimental session and completed a questionnaire to obtain self-reported information about demographics, symptoms and psychological measures. Participants received either course credit or $35.00 for their time.

### The force-matching task

A detailed description of the force-matching device design, functionality and task can be found in McNaughton et al.^29^.

#### Participant positioning

Participants sat at a table and placed their right index finger, just superior to the distal interphalangeal joint. An ergonomic wrist and forearm support system was used to improve comfort in maintaining sustained wrist supination and to avoid any unwanted arm or finger movement.

#### Task

The force-matching task consisted of two conditions: (a) The direct condition, in which participants match a target force by pressing directly on top of a lever, mechanically transmitting the force to the right index finger; and (b) the slider condition, in which participants match the force using their opposite fingertip by moving a slider (potentiometer), controlling the torque motor of the device. Each participant reproduced four target forces (1, 1.5, 2 and 2.5 Newtons) on eight separate trials in a randomised order under both direct and slider conditions, with the order of condition counterbalanced across participants.

During each condition, the device exerted one of the four constant target forces for 3 s. After 2 s of rest, a “go” signal instructed the participants to start matching the target force, either by directly pressing with their left index finger (direct) or by moving the external potentiometer with their left finger (slider). A “stop” signal marked the end of the trial. A force sensor at the end of the lever measured the matched forces applied to the right finger. The participant’s mean matched force was then calculated over the 2.5–3.0 s time interval after the go signal.

### Measures

#### Pain description, Brief Pain Inventory (BPI) and symptom reporting

All chronic pain subjects were asked the location (present or not across different body areas), frequency (present once a month, more than once a month, once a week, more than once a week or daily), chronicity (the year of pain onset) of pain and the self-reported cause of their pain. The pain intensity section of the BPI consists of four items that are scored from 0 (no pain) to 10 (worst possible pain), whereas the functional interference section consists of seven items that are scored from 0 (no interference) to 10 (complete interference)^30^. Reliability coefficients for the BPI severity and interference scales have been reported with alphas ranging from 0.82 to 0.95^31^. Symptom reporting in daily life was determined via the Checklist for Symptom in Daily Life (CSD)^32^. Participants responded to the question ‘To what extent did you experience the following symptoms over the past year?’ on a 5-point scale (never, seldom, sometimes, often, very often), with a total score ranging from 39 (no symptoms) to 195 (high symptom reporter). This scale has been previously used to screen for habitual symptom reporting in daily life, with Cronbach’s alphas ranging from 0.90 to 0.92^33–35^.

#### Self-reported psychological health measures

To investigate correlations with sensory attenuation, four dimensions of psychological health of participants were evaluated: depression, anxiety, state affect and delusional ideology. Depressive symptomology was measured with the ‘9-item Patient Health Questionnaire’(PHQ-9)^36^. Each item on the PHQ-9 is scored from 0 to 3, with a total score ranging from 0 (no depressive symptomology) to 27 (high levels of depressive symptomology). Anxiety was measured with the ‘7-item Generalized Anxiety Disorder Questionnaire’ (GAD-7). Each item on the GAD-7 is scored from 0 to 3, with a total score ranging from 0 (no anxiety) to 21 (high levels of anxiety). Acceptable psychometric properties of the PHQ-9^36^ and GAD-7^37^ are well established. State negative and positive affect were measured with the Positive and Negative Affect Schedule (PANAS), a reliable and well validated instrument^38^ consisting of 10 positive and 10 negative statements^39^. Subjects were asked to indicate the extent their feelings corresponded to the words in the past week on a five-point scale, with a total score ranging from 10 (low negative or positive affect) to 50 (high negative or positive affect). Delusional ideology was measured using the ‘Delusion Inventory’^40^. This consisted of 21 statements in which participants had to respond using a “yes/no” binary scale. This was designed to quantify delusion-like ideas in the general population. A total score was calculated (0–21) with high scores reflecting high levels of delusional ideology.

### Statistical analyses

A number of metrics reflecting the level of sensory attenuation were calculated. Whilst these indices are conceptually similar, they are mathematically different and primarily facilitate comparisons with other findings. In each condition and force, the mean force error (mean matched force minus the target force) and ratio (mean matched force divided by the target force) were calculated. We then calculated composites scores by aggregating the error and ratio values across force levels. This gives a single error or ratio value in each condition for an overall comparison of sensory attenuation between conditions and groups. A further variable was determined via subtracting the error in the slider condition from the error in the direct condition. This provides a single measure of the sensory attenuation relative to the subjects’ performance on the slider, which serves as a reference in which limited (or no) sensory prediction occurs. Thus, this variable quantifies the individual degree of sensory prediction utilised in the task and can be correlated with psychological and symptom questionnaires^41^. All analyses were performed using STATA v16^42^.

To determine any differences of sensory attenuation between the chronic pain and control groups, several analyses were conducted. To determine differences in slope and intercept of the matched forces across force levels we conducted a bootstrapped mixed-effect multilevel regression analysis. Moderation by group (control or chronic pain) was then assessed via the statistical interaction of the above mixed-effect multilevel regression model. Wilcoxon-rank sum analyses were used to determine differences in the magnitude of composite sensory attenuation variables (error, ratio, and sensory prediction) between the chronic pain and control groups. Non-parametric Spearman correlations were used to measure the association between sensory attenuation variables (error, ratio, and sensory prediction) with several self-reported psychological and symptom measures. Finally, Levene’s test was used to determine equality of variance of the composite sensory attenuation variables (error, ratio, and sensory prediction) between the control and chronic pain groups.

During the review process several methodological questions were raised. These specifically were whether the location or the aetiology (known or unknown) of the individuals’ pain influenced the level of sensory attenuation. Post hoc and bootstrapped mixed-effect multilevel regression analyses were conducted with the mean force error as the dependant variable, with the location of pain or aetiology of pain (known or unknown) as the independent variables. These analyses were conducted for both the direct and slider conditions and reported in the supplementary material.

### Ethics approval

The study was approved by the Macquarie University Human Sciences Ethics Subcommittee (Approval number: 52019574612789).

## Results

One-hundred and thirty-one participants were recruited for the study (50% with chronic pain). All completed the force-matching task and questionnaires. Table 1 displays the demographics of all subjects while Table 2 displays the pain profiles of those experiencing chronic pain. Based on established numerical rating scale cut points^43^, this community chronic pain sample reported moderate pain intensity and activity interference. No differences were identified with respect to age, gender, or delusional ideation between the two groups. However, the chronic pain individuals experienced lower positive affect and higher levels of anxiety, depression, physical symptom count and negative affect.

**Table 1:** Subject demographics, psychological covariates, and symptom profile.

	Chronic pain (n = 66)	Control (n = 65)	Difference
Gender (female)	48 (72%)	43 (66%)	$X$^2^ = 0.67, p = 0.41
Age	24.95 (8.86)	23.88 (8.74)	t = 0.70, p = 0.49
Anxiety	7.15 (5.54)	4.06 (3.95)	t = − 3.67, p = 0.0004
Depression	8.58 (6.30)	4.62 (4.64)	t = − 4.09, p = 0.0001
Symptom count	86.83 (28.43)	64.43 (17.75)	t = − 5.40, p < 0.0001
Delusional Ideation	5.78 (4.06)	4.71 (3.39)	t = − 1.64, p = 0.10
Positive affect	27.36 (8.23)	30.69 (8.01)	t = 2.35, p = 0.02
Negative affect	22.02 (8.79)	18.46 (18.47)	t = − 2.63, p = 0.01

Anxiety = GAD-7 (0–21), depression = PHQ-9 (0–27), symptom count = CSD (39–195), delusional ideation = PDI-21 Scale-21 (0–21), and positive/negative affect = PANAS (10–50). Scores above 10 in both the GAD-7 and PHQ-9 are considered to be in the clinical range^36,52^. High habitual symptom reporters are considered to have scores above 100 on the CSD^53^. Healthy PANAS data reflects high positive affect (mean = 40.0/SD = 3.4) and low negative affect (mean = 13.9/SD = 2.4)^54^.

**Table 2:** Chronic pain profile (N = 66).

**Pain location**	
Head, face, or mouth	36 (54.5%)
Neck, back or shoulders	49 (74%)
Arms, Forearms or Hands	23 (35%)
Low back, pelvis, or sacrum	42 (64%)
Legs, knees, or feet	25 (38%)
Abdomen	25 (38%)
**Pain frequency (at least 1 day/week)**	
Head, face, or mouth	22 (61.1%)
Neck, back or shoulders	33 (67.35%)
Arms, forearms or Hands	10 (43.48%)
Low back, pelvis, or sacrum	25 (55.92%)
Legs, knees, or feet	17 (68%)
Abdomen	13 (52%)
**Pain duration (months)**	
Head, face, or mouth	61.13 (62.27)
Neck, back or shoulders	82.24 (83.68)
Arms, forearms or hands	51.95 (54.21)
Low back, pelvis, or sacrum	78.6 (69.33)
Legs, knees, or feet	57.72 (54.42)
Abdomen	73.63 (72.1)
**Pain cause**	
Accident	1 (1.5%)
From work	7 (11%)
Surgical/medical treatment	3 (5%)
Result of illness	9 (14%)
No reason, just developed	31 (47%)
Other	15 (23%)
Average number of pain locations	3.03 (1.4)
Worse pain (0–10)	5.85 (2.02)
Best pain (0–10)	2.14 (1.88)
Average pain (0–10)	4.26 (1.71)
Present pain (0–10)	2.71 (2.31)
Medication relief (0–10)	4.87 (3.2
Activity interference (0–10)	3.56 (2.57)
Mood interference (0–10)	4.77 (3.08)
Walking interference (0–10)	2.55 (2.75)
Work interference (0–10)	3.47 (2.77)
Relationship interference (0–10)	2.65 (3.23)
Sleep interference (0–10)	4.35 (3.09)
Enjoyment interference (0–10)	3.46 (3.03)

Information gathered from location, frequency, duration, and Brief Pain Inventory. Some individuals experienced multiple pain locations, and therefore will have duration and frequency data for multiple pain sites. Pain frequency refers to those experiencing the specific pain location at a rate of at least once per week. Established NRS cut points for worst pain are 1–4 (mild), 5–6 (moderate) and 7–10 (severe)^43^.

### Matched force over differing force levels and effect modification by group

Table 3 displays the mean matched force and standard deviations by target force, condition, and group. In the chronic pain group, the matched force for the direct condition varied significantly across force levels (${\chi }^{2}$(3) = 2136.68, p < 0.001) and a minor flattening of the matched force was observed as the force level increased (ß = 0.96, Z = 44.94, p < 0.001); in the slider condition the matched force similarly varied significantly across force levels (${\chi }^{2}$(3) = 3360.27, p < 0.001), however, a more pronounced flattening of the matched force was observed as the force level increased (ß = 0.88, Z = 55.66, p < 0.001).

**Table 3:** Mean matched force differentiated by group, condition, and force level.

Target force (N)	Chronic pain		Control	
	Direct	Slider	Direct	Slider
1	1.58 (0.71)	0.92 (0.27)	1.38 (0.44)	0.85 (0.23)
1.5	2.10 (0.81)	1.34 (0.34)	1.94 (0.62)	1.26 (0.26)
2	2.58 (0.97)	1.74 (0.40)	2.43 (0.70)	1.63 (0.32)
2.5	2.94 (0.91)	2.05 (0.50)	2.80 (0.79)	1.95 (0.38)

Participants matched each target force on 8 separate occasions. The above represents the means and standard deviations of the matched force aggregated across each force level.

In the control group, the matched force in the direct condition varied significantly (${\chi }^{2}$(3) = 1760.73, p < 0.001) and a minor flattening of the matched force occurred (ß = 0.98, Z = 41.61, p < 0.001). The slider condition similarly varied significantly between force levels (${\chi }^{2}$(3) = 4234.17, p < 0.001), however a more pronounced flattening of the matched force was observed as the force levels increased (ß = 0.86, Z = 63.45, p < 0.001). In both the direct and slider conditions, no evidence of effect modification by chronic pain or control group status was identified; direct condition ${\chi }^{2}$(3) = 0.49, p = 0.92 and slider condition ${\chi }^{2}$(3) = 0.*96, p* = 0.81.

### Force-matching mean error, ratio, and sensory prediction differences between chronic pain and control groups

Table 4 displays the mean error, ratio and sensory prediction values averaged over the target forces, by condition and group. In the chronic pain group, subjects exhibited a higher and less accurate mean force error compared to the control group, however this difference did not reach statistical significance. In the slider condition the chronic pain subject exhibited a more inaccurate underestimation of the target force, compared to the control group, however this difference did not reach statistical significance.

**Table 4:** Force-matching task results (force error, ratio, and sensory prediction) for chronic pain and control groups (means and standard deviations).

	Chronic pain	Control	Group difference
Error (direct)	0.55 (0.81)	0.39 (0.58)	Z = − 0.90, p = 0.37
Error (slider)	− 0.24 (0.33)	− 0.33 (0.25)	Z = − 1.41, p = 0.16
Ratio (direct)	1.36 (0.50)	1.25 (0.34)	Z = − 0.98, p = 0.33
Ratio (slider)	0.88 (0.19)	0.82 (0.15)	Z = − 1.46, p = 0.15
Prediction	0.32 (1.00)	0.06 (0.66)	Z = − 1.27, p = 0.21

Error and ratio values were determined by averaging across the four target forces. The sensory prediction value was calculated by subtracting the mean error in the slider condition from that of the direct condition, indicating the degree of sensory prediction.

### Correlations of force-matching error, ratio, and sensory prediction variables with self-reported psychological measures

There was a significant relationship between force-matching error values and depression symptoms, physical symptom count and positive affect (Table 5), with the slider condition. Scatterplots of these associations are provided in the supplementary material to aid interpretation. Further, this was only observed in the chronic pain group and not the control group. The inverse effect was observed with respect to positive affect, with increasing positive effect relating to a more accurate estimation of the target force in the slider condition. This was similarly seen in the correlations with the ratio values and no correlations were identified using the sensory prediction values (see Supplementary Material).

**Table 5:** Correlations of force-matching (direct and slider conditions) with self-reported psychological measures (reported as mean error spearman correlation coefficients).

	Chronic pain		Control	
	Direct (rho)	Slider (rho)	Direct (rho)	Slider (rho)
Anxiety	0.02, p = 0.84	− 0.19, p = 0.13	0.04, p = 0.77	− 0.21, p = 0.09
Depression	0.06, p = 0.61	− 0.24, p = 0.05	0.05, p = 0.70	− 0.13, p = 0.31
Symptoms	0.17, p = 0.17	− 0.25, p = 0.04	0.05, p = 0.67	− 0.09, p = 0.49
Delusion	0.13, p = 0.29	− 0.09, p = 0.46	− 0.08, p = 0.54	− 0.10, p = 0.41
Positive affect	0.13, p = 0.28	0.28, p = 0.02	− 0.20, p = 0.12	− 0.08, p = 0.52
Negative affect	0.03, p = 0.80	− 0.18, p = 0.15	− 0.03, p = 0.81	− 0.07, p = 0.58

Anxiety = GAD-7 (0–21), depression = PHQ-9 (0–27), symptom count = CSD (39–195), delusional ideation. = PDI-21 Scale-21 (0–21), and positive/negative affect = PANAS (10–50).

### Force-matching error, ratio, and sensory prediction variance differences between chronic pain and control groups

**Figure 1:**
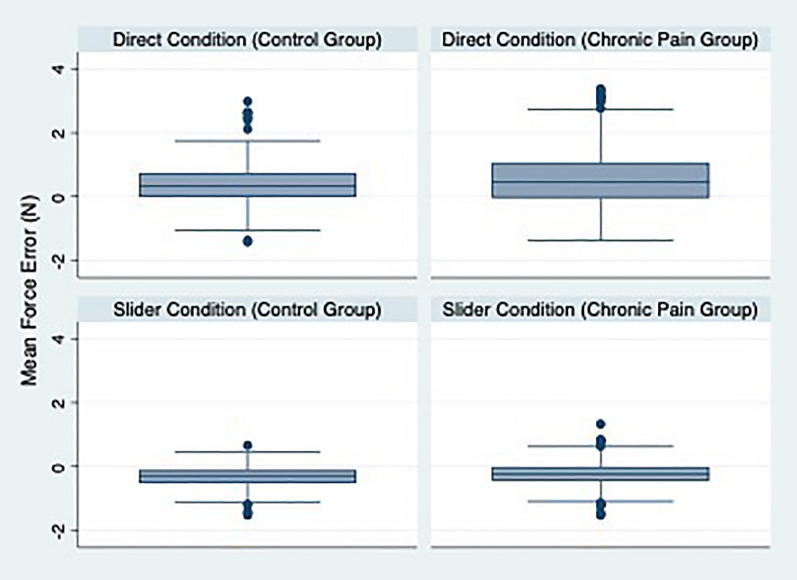
Box plots displaying mean force-matching error (in N) for chronic pain and control groups, and for the two force-matching conditions: direct and slider.

In the direct condition, the chronic pain group exhibited a statically significant increased variance of mean force error: F(1, 129) = 7.22, p = 0.008. This was similarly seen in the slider condition, with some evidence of the chronic pain group experiencing a statically significant increased variance of mean force error: F(1, 129), p = 0.05. Figure 1 displays standard box plots by condition and group and highlights this difference in mean force error variance. With respect to the ratio, there was evidence of a statistically significant difference of variance in the direct (F(1, 129) = 8.25, p = 0.004) and slider (F(1, 129) = 3.82, p = 0.05) conditions. A further increase in variance was observed in the sensory prediction variable F(1, 129) = 10.19, p = 0.002.

## Discussion

This study sought to investigate the sensory attenuation phenomena in subjects with chronic pain, using an experimental paradigm known as the force-matching task. This task is conceptualised within a cognitive model known as Predictive Processing, and our work further examines the relevance of this model in chronic pain symptoms. Sensory attenuation is essentially the turning up (or down) on the gain of a specific sensory channel and we hypothesised this may underlie attentional biases identified in some individuals with chronic pain. Overall, our findings reveal no clear differences in the magnitude of sensory attenuation between groups. However, we did identify a greater variance of the error, ratio, and sensory prediction variables in those with chronic pain, as well as subtle correlations between the sensory attenuation metrics and psychological measures.

Both individuals with chronic pain and healthy controls were found to overestimate the matched force in the direct condition, demonstrating the normal sensory attenuation phenomenon. This contrasts with a more accurate estimation of the target force identified in the slider condition, which is most likely due to a reduction of predictive efference copy signals leading to the increased weighting of sensory feedback. This lack of differentiation of sensory attenuation between the chronic pain subjects and controls is a novel finding and may highlight the normal predictive strategies used in the processing of non-noxious sensorimotor stimuli.

One explanation of the absence of differentiation between chronic pain or controls may be due to the location of the task relative to the pain symptoms reported. Whilst post hoc analyses revealed no differences in relation to pain location, there is evidence to suggest pain-related attentional effects on the processing of sensory inputs are sensitive to the body location which is in pain^44^. We also note a high level of overlapping pain locations, which may have obscured differences in our sample. Similarly, reduced perceptual discrimination and tactile acuity has been more readily observed in the body regions affected by chronic pain, such as in individuals with complex regional pain syndrome^45^, chronic upper limb neuropathic pain^46^ and chronic back pain^47^. A recent study highlighting a similar paradigm identified while matching a target force using the extensor musculature of the low back, chronic back pain individuals experienced an increased error. It is important to highlight the mean maximum voluntary contraction was higher (> 40%) in that experimental paradigm, which has been shown to diminish sensory attenuation effects^48^. Notwithstanding, it would be reasonable to further test this device in those with localised chronic pain symptoms in the arm or hand, to determine whether the location of the symptoms impact on sensory attenuation effects.

Similarly, a lack of differences in the magnitude of sensory attenuation in chronic pain individuals may have also occurred due to our stimuli not reflecting (or activating) specific nociceptive sensory inputs. Within a Predictive Processing framework much of the previous explanations in chronic pain^3^ have activated nociceptive sensory channels or manipulated pain related priors. Therefore, the specificity of the task in the sensorimotor domain may have a limited influence the on the pain experience.

Whilst no differences in the magnitude of sensory attenuation were identified between chronic pain and control groups, individuals with chronic pain did experience an increased variability of sensory attenuation particularly in the direct condition. A likely explanation for this result is heterogeneity of our sample of individuals with chronic pain. Individual differences related to pain and sensory experiences are well documented^1^, and this is further highlighted by Hoskin et al.^49^ within a predictive processing framework, who identified individual differences related to stimuli expectancies when manipulating model parameters (such as sensitivity to aspects of pain cues). Whilst all chronic pain subjects experience persistent pain, the exact mechanisms are likely to differ from individual to individual. Clinically, this may be observed by some individuals being more sensory dominant, responding well to somatosensory training (such as biofeedback), while others may be more psychologically dominant, whereby targeting information-processing biases may have greater therapeutic benefits. Our results suggest that this range of sensory prediction strategies would be of value to investigate further.

Previous correlational studies between the sensory attenuation variables with various self-reported constructs have resulted in mixed results. For example, deficits in sensory prediction have been related to delusional ideation in a community sample^41^, however absence of this relationship has also been reported^50^. Using the same scale in our study, we identified no relationship between delusional ideation and sensory attenuation. We did, however, identify small correlations between the matched force error with depressive symptoms and physical symptoms. Specifically, there was a correlation in the slider condition, between greater underestimation of the target force with high depressive symptoms and symptom count. The inverse relationship was identified with increasing positive affect, and this was only observed in the chronic pain group, indicating a potential relationship between sensorimotor and emotional processing. There is experimental evidence to suggest an upregulated relationship with cortical regions responsible for emotional processing and somatosensory integration in some individuals with chronic pain^51^ and our results may further highlight this processing in non-noxious stimuli. It is presently unclear why this only occurred in the slider condition, and this warrants further investigation. Considerations regarding this finding include the modest effect sizes and statistical significance levels, as well as the observed relationships are identified in the condition with less between subject variation, and this may represent a better signal–noise ratio compared to that in the direct condition.

Some limitations of the current study are as follows. The chronic pain sample were effectively a community sample, rather than a clinical sample, and their present pain levels were relatively low. It is possible that different results would be observed in those with chronic pain who were in clinical care and experiencing higher pain intensity. We recruited a pain sample independent of pain aetiology or location. This was done because it was hypothesised sensory attenuation could represent a shared central neurobiological process related to attentional biases. Future research utilising this task should investigate the sensory attenuation phenomenon in specific pain phenotypes, specifically those with nocioplastic, neuropathic or inflammatory pain. Further, more detailed information regarding the pain diagnoses, pain locations and whether the pain is stimulus evoked or ongoing/spontaneous would aid in determining the relevance of the sensory attenuation phenomenon in subgroups of chronic pain. The cross-sectional design of this study does not enable investigation of temporality of the observed effects; therefore, we do not know if the differences and correlations identified in the chronic pain group are predisposing or a sequala of chronic pain. It has been suggested that differences in force matching device design and functionality may lead to differences in results^29^, we hope that our previous research publishing the device software and blueprints aids in replicability of this research.

Whilst no differences in the magnitude of sensory attenuation were observed, we have demonstrated that this sample of people with chronic pain exhibit a more variable tactile reproduction of a target force compared to healthy controls. Further, force estimation in the slider condition was correlated with self-reported psychological and symptom measures, potentially indicating a relationship between sensory and emotional processing. This study provides evidence of minimal differences in sensory prediction strategies, measured via the force-matching task, utilised by those with chronic pain. Future research is needed to determine the relevance of sensory attenuation in neuro-cognitive models related to symptom perception.

## Supplementary Information

Supplementary Information.

## Data Availability

The datasets generated during and/or analysed during the current study are not publicly available due to ethical restrictions, however further analyses may be completed by the authors on reasonable request.

## References

[1] Fillingim RB (2017). Individual differences in pain: Understanding the mosaic that makes pain personal. Pain.

[2] Stilwell P, Harman K (2019). An enactive approach to pain: Beyond the biopsychosocial model. Phenomenol. Cogn. Sci..

[3] Ongaro G, Kaptchuk TJ (2019). Symptom perception, placebo effects, and the Bayesian brain. Pain.

[4] Henningsen P (2018). Persistent physical symptoms as perceptual dysregulation: A neuropsychobehavioral model and its clinical implications. Psychosom. Med..

[5] Van den Bergh O (2017). Symptoms and the body: Taking the inferential leap. Neurosci. Biobehav. Rev..

[6] Clark A (2013). Whatever next? Predictive brains, situated agents, and the future of cognitive science. Behav. Brain Sci..

[7] Seth AK, Friston KJ (2016). Active interoceptive inference and the emotional brain. Philos. Trans. R. Soc. B Biol. Sci..

[8] Friston K (2005). A theory of cortical responses. Philos. Trans. R. Soc. B Biol. Sci..

[9] Wolpe N (2016). Ageing increases reliance on sensorimotor prediction through structural and functional differences in frontostriatal circuits. Nat. Commun..

[10] Blakemore SJ, Wolpert DM, Frith CD (2002). Abnormalities in the awareness of action. Trends Cogn. Sci..

[11] Shergill SS (2003). Two eyes for an eye: The neuroscience of force escalation. Science.

[12] Bays PM, Wolpert DM, Flanagan JR (2005). Perception of the consequences of self-action is temporally tuned and event driven. Curr. Biol..

[13] Wolpert DM, Diedrichsen J, Flanagan JR (2011). Principles of sensorimotor learning. Nat. Rev. Neurosci..

[14] Blakemore SJ, Wolpert DM, Frith CD (1998). Central cancellation of self-produced tickle sensation. Nat. Neurosci..

[15] Bansal S, Ford JM, Spering M (2018). The function and failure of sensory predictions. Ann. N. Y. Acad. Sci..

[16] Schoth DE, Nunes VD, Liossi C (2012). Attentional bias towards pain-related information in chronic pain; A meta-analysis of visual-probe investigations. Clin. Psychol. Rev..

[17] Tabor A (2017). Pain: A statistical account. PLoS Comput. Biol..

[18] Brown H (2013). Active inference, sensory attenuation and illusions. Cogn. Process..

[19] Parees I (2014). Loss of sensory attenuation in patients with functional (psychogenic) movement disorders. Brain.

[20] Shergill SS (2005). Evidence for sensory prediction deficits in schizophrenia. Am. J. Psychiatry.

[21] Voss M (2007). An improvement in perception of self-generated tactile stimuli following theta-burst stimulation of primary motor cortex. Neuropsychologia.

[22] Wolpert DM, Flanagan JR (2001). Motor prediction. Curr. Biol..

[23] Shergill SS (2014). Functional magnetic resonance imaging of impaired sensory prediction in schizophrenia. JAMA Psychiat..

[24] Hechler T, Endres D, Thorwart A (2016). Why harmless sensations might hurt in individuals with chronic pain: About heightened prediction and perception of pain in the mind. Front. Psychol..

[25] Palmer CE, Davare M, Kilner JM (2016). Physiological and perceptual sensory attenuation have different underlying neurophysiological correlates. J. Neurosci..

[26] Nijs J (2021). Central sensitisation in chronic pain conditions: Latest discoveries and their potential for precision medicine. Lancet Rheumatol..

[27] Coppieters MW (2021). Sense of effort is distorted in people with chronic low back pain. Musculoskelet. Sci. Pract..

[28] Petersen S (2014). Categorical interoception: Perceptual organization of sensations from inside. Psychol. Sci..

[29] McNaughton D (2021). Design, development and functionality of a haptic force-matching device for measuring sensory attenuation. Behav. Res. Methods.

[30] Cleeland CS, Ryan KM (1994). Pain assessment: Global use of the Brief Pain Inventory. Ann. Acad. Med. Singap..

[31] Keller S (2004). Validity of the brief pain inventory for use in documenting the outcomes of patients with noncancer pain. Clin. J. Pain.

[32] Wientjes CJ, Grossman P (1994). Overreactivity of the psyche or the soma? Interindividual associations between psychosomatic symptoms, anxiety, heart rate, and end-tidal partial carbon dioxide pressure. Psychosom. Med..

[33] Han J (2000). Psychosomatic symptoms and breathing pattern. J. Psychosom. Res..

[34] Constantinou E (2013). Inducing symptoms in high symptom reporters via emotional pictures: The interactive effects of valence and arousal. J. Psychosom. Res..

[35] Walentynowicz M (2018). Sensory and affective components of symptom perception: A psychometric approach. J. Exp. Psychopathol..

[36] Kroenke K, Spitzer RL, Williams JB (2001). The PHQ-9: Validity of a brief depression severity measure. J. Gen. Intern. Med..

[37] Kroenke K (2016). The Patient Health Questionnaire Anxiety and Depression Scale (PHQ-ADS): Initial validation in three clinical trials. Psychosom. Med..

[38] Crawford JR, Henry JD (2004). The Positive and Negative Affect Schedule (PANAS): Construct validity, measurement properties and normative data in a large non-clinical sample. Br. J. Clin. Psychol..

[39] Watson D, Clark LA, Tellegen A (1988). Development and validation of brief measures of positive and negative affect: The PANAS scales. J. Pers. Soc. Psychol..

[40] Peters E (2004). Measuring delusional ideation: The 21-item Peters et al. Delusions Inventory (PDI). Schizophr. Bull..

[41] Teufel C (2010). Deficits in sensory prediction are related to delusional ideation in healthy individuals. Neuropsychologia.

[42] StataCorp. *Stata Statistical Software: Release 15.* (StataCorp LLC, 2017).

[43] Atkinson TM (2010). The brief pain inventory and its “pain at its worst in the last 24 hours” item: Clinical trial endpoint considerations. Pain Med..

[44] Vanden Bulcke C (2013). The anticipation of pain at a specific location of the body prioritizes tactile stimuli at that location. PAIN®..

[45] Moseley GL, Zalucki NM, Wiech K (2008). Tactile discrimination, but not tactile stimulation alone, reduces chronic limb pain. PAIN®..

[46] Taylor KS, Anastakis DJ, Davis KD (2010). Chronic pain and sensorimotor deficits following peripheral nerve injury. PAIN®..

[47] Stanton TR (2017). Feeling stiffness in the back: A protective perceptual inference in chronic back pain. Sci. Rep..

[48] Walsh LD, Taylor JL, Gandevia SC (2011). Overestimation of force during matching of externally generated forces. J. Physiol..

[49] Hoskin R (2019). Sensitivity to pain expectations: A Bayesian model of individual differences. Cognition.

[50] Humpston CS (2017). Evidence of absence: No relationship between behaviourally measured prediction error response and schizotypy. Cogn. Neuropsychiatry.

[51] Bushnell MC, Ceko M, Low LA (2013). Cognitive and emotional control of pain and its disruption in chronic pain. Nat. Rev. Neurosci..

[52] Spitzer RL (2006). A brief measure for assessing generalized anxiety disorder: The GAD-7. Arch. Intern. Med..

[53] Bogaerts K (2010). Negative affective pictures can elicit physical symptoms in high habitual symptom reporters. Psychol. Health.

[54] Sibille KT (2012). Affect balance style, experimental pain sensitivity, and pain-related responses. Clin. J. Pain.

