# Supplementary material for: Perceptual sensory attenuation in chronic pain subjects and healthy controls
Source: Sci Rep. 2022 May 27;12:8958. doi: 10.1038/s41598-022-13175-4 (PMC9142587; doi:10.1038/s41598-022-13175-4)
Supplement: Supplementary file 1 — Supplementary Information. [file 41598_2022_13175_MOESM1_ESM.docx]

**Supplementary Material**

Table 1. Correlations of force-matching (direct and slider conditions) with self-reported psychological measures (reported as mean ratio spearman correlation coefficients)

|  | Chronic Pain | | Control | |
| --- | --- | --- | --- | --- |
|  | Direct (*rho)* | Slider (*rho)* | Direct (*rho)* | Slider (*rho)* |
| Anxiety | 0.01, p = .88 | -0.21, p = .08 | 0.05, p = .68 | -0.25, p = .04 |
| Depression | 0.05, p = .68 | **-0.27, p = .03** | 0.05, p = .71 | -0.16, p = .17 |
| Symptoms | 0.16, p = .18 | **-0.27, p = .03** | 0.05, p = .71 | -0.10, p = .41 |
| Delusion | 0.12, p = .32 | -0.07, p = .56 | -0.06, p = .61 | -0.13, p = .28 |
| Positive affect | 0.12, p = .30 | **0.29, p = .01** | -0.17, p = .17 | -0.05, p = .70 |
| Negative affect | 0.02, p = .85 | -0.20, p = .09 | 0.001, p = .99 | -0.12, p = .36 |

Table 2. Correlations of force-matching prediction values with self-reported psychological measures (reported as mean sensory prediction value spearman correlation coefficients)

|  | Chronic Pain (*rho)* | Control (*rho)* |
| --- | --- | --- |
| Anxiety | -0.04, p = .77 | -0.04, p = .73 |
| Depression | -0.03, p = .80 | 0.001, p = .99 |
| Symptoms | 0.06, p = .65 | 0.012, p = .90 |
| Delusion | 0.07, p = .57 | -0.10, p = .41 |
| Positive affect | 0.22, p = .08 | -0.22, p = .07 |
| Negative affect | -0.05, p = .71 | -0.03, p = .80 |

Figure 1. Scatterplot displaying the relationship between the mean force error and depressive symptoms (measured via the PHQ-9) in the slider condition.

Figure 2. Scatterplot displaying the relationship between the mean force error and symptom count (measured via the CSD) in the slider condition.

Figure 3. Scatterplot displaying the relationship between the mean force error and positive affect (measured via the PANAS) in the slider condition.

**Post hoc analyses**

During the review process several methodological questions were raised. These were specifically to determine whether the location or the aetiology (known or unknown) of the individuals’ pain influenced the level of sensory attenuation.

*Location of pain*

Subjects were asked to indicate the location of their pain into 6 categories:

1. Head, face, or mouth
2. Neck, back or shoulders
3. Arms, Forearms or Hands
4. Low back, pelvis, or sacrum
5. Legs, knees, or feet
6. Abdomen

A post hoc bootstrapped mixed-effect multilevel regression analyses were conducted with the mean force error as the dependant variable with the location of pain as the independent variable. This was conducted for both the direct and slider conditions. The results were statistically non-significant in both the direct ($\chi^{2}$(5) = 0.02, p > .999) and slider conditions ($\chi^{2}$(5) = 0.06, p > .999). It is important to note these categories were not mutually exclusive and a number of individuals experienced multiple areas of pain.

In addition, to further determine if location of pain proximal to the site of testing influenced mean differences in the force-matching task, individuals who reported pain in the arms, forearms or hands were excluded from the primary analyses (N=23). No statistically significant differences were identified compared to the main conclusions of the manuscript (see Table 3).

*Table 3.*

*Force-matching task results (force error, ratio, and sensory prediction) for chronic pain and control groups (means and standard deviations). Subjects with pain in the arm, forearm or hand (N=23) removed from Chronic pain group*

|  | **Chronic Pain** | **Control** | **Group Difference** |
| --- | --- | --- | --- |
| Error (Direct) | 0.63 (0.78) | 0.39 (0.58) | Z = -1.51, p = .12 |
| Error (Slider) | -0.21 (0.32) | -0.33 (0.25) | Z = -1.63, p = .10 |
| Ratio (Direct) | 1.41 (0.50) | 1.25 (0.34) | Z = -1.51, p = .13 |
| Ratio (Slider) | 0.90 (0.19) | 0.82 (0.15) | Z = -1.83, p = .07 |
| Prediction | 0.41 (0.91) | 0.06 (0.66) | Z = -1.78, p = .08 |

*Pain aetiology (known or unknown)*

Subjects were asked regarding the aetiology of their pain reported from a medical practitioner. Forty-seven percent of the sample indicated an unknown cause of their pain. A post hoc bootstrapped mixed-effect multilevel regression analyses were conducted with the mean force error as the dependant variable with the aetiology of pain as the independent variable. These were conducted for both the direct and slider conditions. The results were statistically non-significant in the direct ($\chi^{2}$(1) = 0.13, p > .72) and slider conditions ($\chi^{2}$(1) = 1.12, p > .29).
